# Psychosocial rehabilitation experiences of women victims of armed conflict in Montes de María, Colombia

**DOI:** 10.1186/s13690-021-00548-w

**Published:** 2021-03-10

**Authors:** Laura Camila Sarmiento-Marulanda, Amira Ayleen Aguilera-Char, Catalina González-Gil, Wilson López-López

**Affiliations:** 1 Psychology Faculty and Head of Social and Organizational Psychology, Universidad de La Sabana, Campus Universitario del Puente del Común, Km. 7. Autopista Norte de Bogotá, Chia, Colombia; 2 Law, Political and Social Sciences Faculty, Universidad La Gran Colombia, Bogotá, Colombia; 3 Psychology School, Universidad de La Sabana, Chia, Colombia; 4 Psychology Faculty, Pontificia Universidad Javeriana, Bogotá, Colombia

**Keywords:** Psychosocial, armed conflict, Social intervention, Social policy, Victims, Integral reparation, Colombia

## Abstract

**Background:**

After 9 years of the ground-breaking social policy Law 1448 of 2011 -Victims Law- and its extension until 2030, the Colombian State and other stakeholders have made several efforts towards granting the right of integral reparation for more than 9 million victims that are recognized in the Colombian transitional context. Psychosocial rehabilitation is a Victims Law’s reparation measure whose objective is to re-establish the psychosocial, physical and mental health welfare in the individual, familiar and community levels. This study aims to understand the experiences of psychosocial rehabilitation of women victims of armed conflict in Montes de Maria and the underlying social intervention paradigms that guide the Law’s implementation.

**Methods:**

Based on a qualitative design with a phenomenological approach, narrative tools and thematic network analysis permitted to give voice to the women participants. Individual narrative interviews were conducted with 12 women victims and a focus group with eight of them was used as a triangulation strategy.

**Results:**

Although the Victims Law is oriented by a sociopolitical intervention paradigm, the stories of the women’s victims of Montes de María mainly evidenced non-sociopolitical interventions with humanitarian assistance towards revictimization and State abandonment. As a coping mechanism towards the State negligence encountered, women strive to overcome psychosocial trauma by developing agency and community resources for the resignification of the traumatic experiences and peacebuilding.

**Conclusions:**

For the Victims Law to achieve its integrality aim, the psychosocial approach should be implemented through all its measures but remains absent in Montes de Maria. The diversity of victim’s individual and collective initiatives that were found, can contribute towards transformative and participatory psychosocial intervention with community’s resources. Women victims can perform as advisors and collaborators in the implementation of individual and collective reparation, which remains as an opportunity for psychosocial rehabilitation and peacebuilding. Further monitoring and evaluation of the law with a territorial and differential perspective is required to respond to the victim’s needs.

## Background

The Colombian armed conflict involves a significant social tissue fracture after more than 60 years of violence. Victims rise to 9,005,319 million which corresponds to 25% of the country’s population [1]. Structural problems connected with inequity and marginalization, amongst others, have been delayed and unsolved by the different stakeholders [2–5]. Physical and mental health, as well as psychosocial welfare affectations, including the sense of community and prosocial behavior, reflect damage due to the permanent exposition to violence [6–10]. Such oppression towards Colombians citizenship [11–13] constitutes an urgent State intervention matter. Psychosocial rehabilitation (PR) has therefore become a co-responsible process in Colombian Social policy to improve public health and successfully grant the victim’s well-being towards integral reparation.

In this order, the Victims Law (VL) 1448/11 regulated by the Decree 4800/11, seeks to provide prevention, assistance and protection for the victims’ rights of truth, justice and reparation based on administrative, social, judicial and economic measures at individual and collective levels [2, 4]. It was elaborated through a collective process with more than 4000 victims, social leaders and representatives of different social organizations, presented in nine congressional audiences and cities. This construction process “from below” avoids the decontextualized bias of dominant classes [14]. Also allows a dialogue with the oppressed communities, seeking proper moral and symbolic reparation measures without re-victimization practices. It follows a psychosocial approach that was crucial since its elaboration, and further implementation, through programs, strategies and actions that intend to regain social equilibrium [2, 12, 15]. A process that strengthens personal and collective community resources through active citizen participation for the construction of a more democratic State and Peace [2, 3, 5, 13, 16–18].

The recognition of a participatory social intervention is fundamental from the construction, implementation and monitoring of the VL. It conceives humanitarian, Human Rights, differential, participatory and transformative approaches, recognizing the deep historical roots of violence, inequity and marginalization that produced victimization [2]. In this sense, following Corvalán’s [19] conception on social intervention paradigms which materialize the State’s social policies orientation, socio-political and non-socio-political interventions are found. In this order, socio-political oriented social policies, such as the VL, recognizes major societal objectives as a critic of the development model. Consequently, acting on society’s structural conditions require social transformations in order to build sustainable interventions; as well as avoiding the repetition of the violent acts, for democracy strengthening, forgiveness and reconciliation [2, 14, 17, 19–23]. In response to this necessity, in December 2019, was granted the extension of the VL until 2030 through the Constitutional Court Sentence C-588/19 for the continuation of the reparation process [2]. Furthermore, as interventions in Colombia which are mainly focused on psychosocial support programs lack of continuity due to the armed conflict, results and effectiveness of these programs have not been evaluated, neglecting victims’ reparation towards well-being and quality of life [24]. Therefore, in this Law’s extension context, it becomes relevant to understand women victim’s experiences on PR to contribute to more pertinent and transformative social policy interventions in Colombia.

### Psychosocial approach of the Victim’s Law: psychosocial rehabilitation challenges

In Colombia, a complex paradigm [25] has encountered various theoretical and practical aspects of the psychosocial approach. Including at its base, a community mental health and human rights perspective nurtured from Latin American countries [16, 26, 27]. This approach includes a critical social-community psychology analysis since the actual societal system founded on inequality and social exclusion is unsustainable for the victim’s well-being [5, 27–30]. This sociopolitical context systematically affects the population’s constitutional rights demanding effective reparation. One that does not exclusively rely on the non-sociopolitical or directive interventions towards assistance responses that perpetuates minorities and vulnerable groups marginalization and exclusion, but rather empowers them [2, 3, 14, 16, 19, 27]. Indeed, it is through participation that the National System of Attention and Integral Reparation of the Victims, SNARIV -initialisms in Spanish-, has tried to grant the rights of truth, justice and reparation with material and symbolic measures in a horizontal dialogue between popular and scientific knowledge [6, 14, 31–33].

Regarding PR whose objective is to re-establish physical, mental, psychosocial health and welfare by overcoming the victimizing acts affectations in the individual, familiar and community levels; address structural violence and gives control to victims in the decision-making process [3, 34–36]. In that sense, PR includes actions focused on the victim’s and communitarian resources and agency to overcome victimization rather than emphasizing on psychopathological or deficit approaches that derive from a reductionist individual analysis of armed conflict [3, 15, 16, 35, 37, 38]. The latter one, objectifies victims reducing their personal and collective possibilities of contributing to their reparation process to mere integrative acts remaining in a re-victimizing, negative feedback loop situation [14, 36, 39–41]. On the contrary, agency is therefore a capacity with which victims can transform their situation, beyond such revictimization, intervening with decisions and actions that enables them to resist the dominant power as well as to become the owners of their existence and create their own trajectories as subjects [42].

By intervening at structural levels, PR is founded on a differential approach that prioritizes specialized intervention based on life cycle, disability, ethnicity and gender [2, 35]. Related with the latter, previous works [27, 38, 43–47] emphasize women’s role in response to armed conflict to adjust rehabilitation interventions, that include a gender analysis of psychosocial affectations such as decomposition of their family roles and support networks or institutional dependency [48]. In this sense, based on the VL’s 49 article, a form of psychosocial care for women victims is oriented to restoration of their rights and their autonomy strengthening [2]. This, as a need to generate changes at the structural level regarding the patriarchal order, the recognition of women victim’s needs and the symbolic repercussions of feminine and masculine roles [27, 38, 47]. PR is relevant in reparation processes as it has been proven in different countries, its effects on lessening symptoms at the individual-clinical and psychosocial levels by sharing experiences and agency, improving the way in which women victims relate to their community [24, 49]. It also allows the skills and knowledge consolidation through workshops and training that serve as tools for empowering women in their contexts [15, 49].

However, there are challenges of PR implementation related with integral reparation and non-repetition rights since the armed conflict has not ceased [50], showing a high complexity due to neoliberal conditions of the Colombian State that prevail [51–53]. Victims encounter difficulties for the materialization of fundamental rights in a human rights vulnerability framework; characterized by extreme poverty, lack of access to the guarantee of their basic needs and systematic social leaders homicides; as Colombian State’s historical and structural violence is perpetuated throughout ineffective public policies that do not limit the escalation of social inequality and marginalization with a differential impact on women [24, 27, 34, 44, 47, 50–54]. Furthermore, despite the fact that the VL is a ground-breaking social policy and includes a vast amount of measures that the Colombian State has committed to, remains a gap between the rights it aims to grant and the articulated, institutional and intersectoral actions it assists [26, 35].

Indeed, social intervention in the country has been much more centered in the consequences that generate violence rather than on the understanding of the conflict’s structural roots, driven by a non-sociopolitical intervention paradigm [14, 16, 39]. Framed on a functionalist vision of society based on the search of an equilibrium system by adaptation and accommodation of the damages produced. In such a way, narrowly intervening problems and collectives, but not mobilizing the values and socioeconomic bases in which are distributed society’s resources [14]. Promptness and emergency are characteristics of this type of interventions mostly called as humanitarian [39, 55], but they remain insufficient to resolve structural precariousness and armed conflict in which victims are found [26, 39, 55, 56]. Following compassion policies, humanitarian intervention relies on delayed indemnification, conceiving victims as passive actors, beneficiaries or mentally ill persons [39, 55]. In this context, the quest towards an interventive equilibrium amongst needs satisfaction and reestablishment of rights, as well as the strengthening of political and community development is salient [16].

In relation to the above, the debate on the victim’s social intervention is broadened by the psychosocial perspective towards an attention that truly comprehends human phenomena on a complementary approach between the structural, cultural and subjective dimensions, centered in agency [3, 5, 27]. Therefore, as we recognize the victims’ needs and agency, it is our interest as psychologists to further contribute to the comprehension along with the transformation of the negative psychosocial dynamics that characterize the Colombian armed conflict [2, 21, 26, 27]. We attempt to widen the scope of the project “Women and narratives of reparation: gender category problematizing the Colombian Transitional Justice”, which evidenced the continuity of gender regimes at times of war and peace, as well as the articulation of patriarchal governance with transitional justice frameworks [47]. In this sense, this study aims to understand the PR experiences of women victims towards the VL implementation in Montes de Maria, as well as the coherence between the law’s epistemological background and its implementation, to further contribute to the construction of pertinent and improved PR practices in Colombia.

## Method

### Study design and setting

A qualitative design with a phenomenological approach and an exploratory and interpretive scope was conducted [57, 58], enabling us to understand the experiences of PR of women victims of armed conflict in the implementation of the Right of Integral Reparation of the VL. The municipalities included were El Carmen de Bolívar and San Juan de Nepomuceno in Montes de María, located in the Caribbean region, which is one of the most violent regions in the country’s history due to the presence of various armed actors [59].

### Participants

Individual narrative interviews were conducted with 12 women victims and a focus group with eight of them was used as triangulation. Participants were contacted by a social leader gatekeeper from the Colectivo de Comunicaciones de Montes de María Línea 21 and selected through a snowball method. They were all adult women whose ages ranged from 35 to 70. These women develop farm work and social leadership, have low educational levels and socio-economic vulnerability conditions derived from the armed conflict [44].

### Procedure

At the research’s early stages, women participants had a presentation of the research aims as well as were informed about voluntary participation and the possibility of withdrawing at any time. Participants who understood the research procedures were asked to provide informed consent [60]. The research design, data collection instruments and procedures received ethical approval from the Social Policy graduate program of the University of Brasilia, it took all the ethical precautions recommended by Resolution CNS No. 466/2012 of Brazil’s National Council of Health. Individual narrative interviews were conducted with 12 women in 2017, and eight women participated in a focus group in 2018 as a data collection and triangulation technique, as well as a collaborative effort with the participants, following Caillaud and Flick’s methodological orientation [61]. In both scenarios participants told and discuss their stories in Spanish, placing their experience in an organizing sequence of events, shaping their individual and social life [62–65]. The encounters were recorded, transcribed in Spanish and translated to English. Confidentiality of women’s data was ensured by replacing personal information; we considered ethical criteria from the Deontological and bioethical Psychology Code, as well as the Colombian School of Psychologists and reflected carefully on possible ethical concerns beyond informed consent during the study [60].

We carried out a thematic network analysis, organizing and systematizing qualitative information into basic (BT), organizers (OT), and global themes (GT) following Attride-Stirling [66] orientation. Triangulation of researchers was used [67]. As well as a the mentioned methodological triangulation approach adopted by the use of a focus group as an in-depth interpretation strategy of the data collected through interviews [61]. The women’s interpretations helped to validate the main conclusions established by the researchers. Using NVivo12, the inductive analysis consisted of a permanent and layered movement between data and the successive themes proposed. During the in-depth analysis and triangulation stages, two (GT) emerged: “Experiences of state interventions” and “Experiences of State abandonment”. In this order, the organization comprehends the experiences and meanings attributed by them in relation to PR.

## Results

**Fig. 1:**
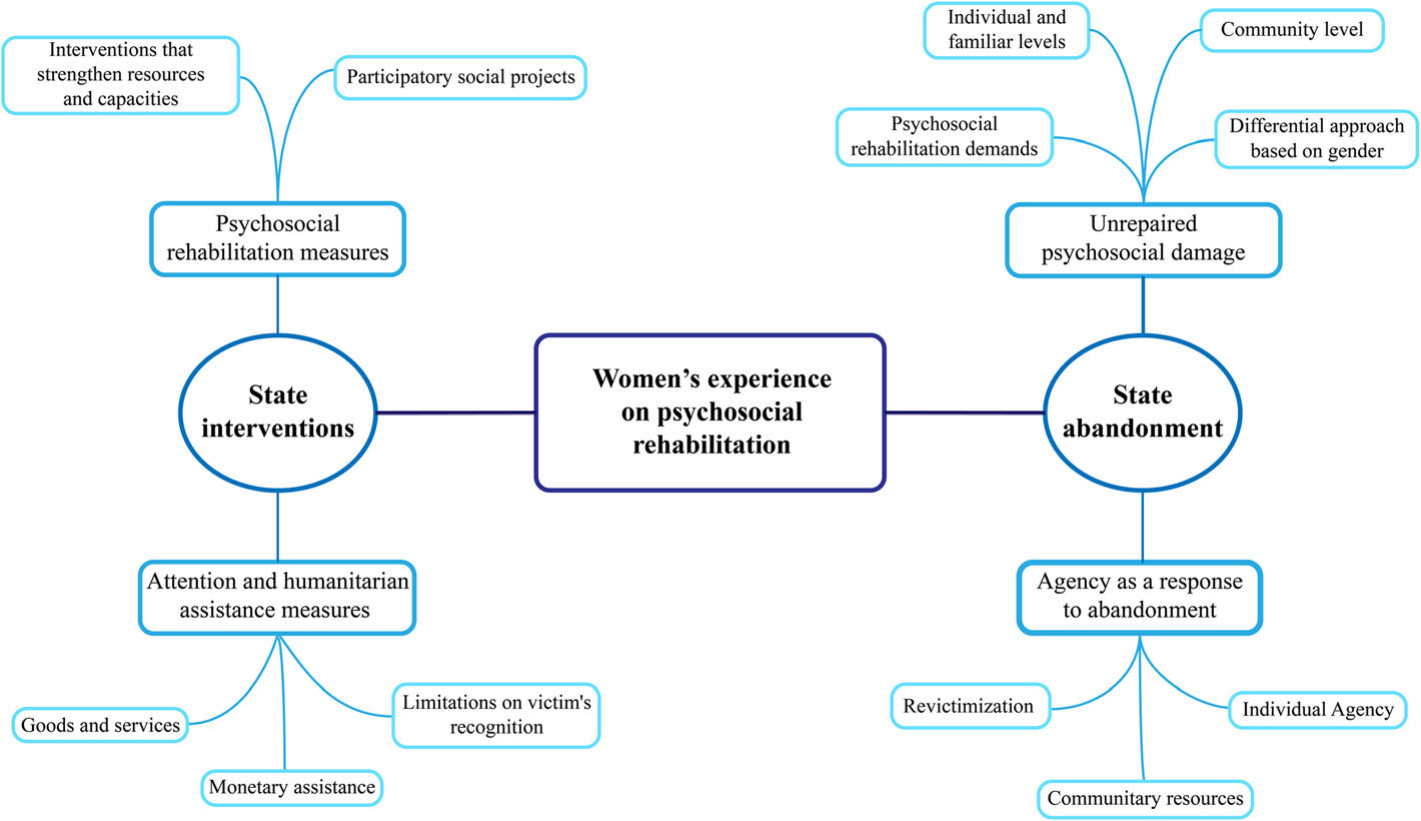
Women’s experience on psychosocial rehabilitation in Montes de Maria, Colombia, 2017–2019

We collected women victims’ voices through narrative tools considering data saturation [68] and the aspects previously described. They were codified building thematic networks (Fig. 1) emerging from women’s shared experiences rather than on individual stories [66]. In this section, we show fragments of the women stories that evidence how they experience disintegrated PR intervention regarding the VL implementation.

### State interventions: humanitarian assistance experiences framed in a (dis) integrated reparation

Results enable us to argue that social intervention is more evidenced by women’s narratives concerning attention and humanitarian assistance measures (OT) instead of integral reparation measures. As VL’s humanitarian interventions do not imply reparation, satisfaction and non-repetition measures through restitution, indemnification or PR, they remain scarce at the implementation level. In relation to reparation measures, although fewer, they are related with indemnification and PR interventions in terms of material, moral and symbolic dimensions. We noticed how in some cases they were beneficiaries of emergency humanitarian assistance but did not experience the psychosocial support or integral reparation stipulated by the law. Also, we found cases without indemnification after 20 years of being forcibly displaced. Women identified monetary grants and, in a few cases, alimentary or clothing aids that were once or a few times received after the victimizing event; humanitarian assistance interventions that do not transform their conditions in the long term [26, 39]. They usually do not identify the institution that provided the humanitarian assistance and feel unaware about the attention and reparation route of the Victim’s Unit.

*“(...) the only thing that I received is humanitarian aid: 210.000 pesos that they gave me one day, that’s it”* (Interview 1).

*“There was no direct manifestation from the State. I remember that my father once brought food, a sack, a bag, it was the only thing I remember. But I don’t remember how he got it, maybe someone in the Red Cross helped us but since then it has been very hard for us.”* (Interview 9).

In these cases, compassion policies, as an element of humanitarianism, is mediating the institutional requirements expressed by the victims [39, 55]. They indicate that in order to access goods and social services related to humanitarian assistance they should exhibit their suffering to be recognized as such. A situation that places limitations toward the recognition of the victim’s human dignity and rights. In some cases, women received monetary humanitarian aids that they regret accepting, as they are still waiting for material, moral and symbolic reparation after many years.

*( … ) for the State the person has to arrive very badly dressed, foul-mouthed and everything, be like an indigent, and the problem is that one wasn’t an indigent, one didn’t live under the bridge, had a family, a lifestyle ( … )* (Interview 1).

*(...) in humanitarian aid, here in Acción Social (Government institution) they gave me one million three hundred eighty thousand pesos in twenty years. I regret receiving them because now I’m still waiting for them to pay my forced displacement, but I call, and they tell me something different each time (...)* (Interview 3).

In other cases, women received an indemnification that responds to the reparation material dimension but remain feeling without integral reparation. This intervention disjointed from the others, does not include a human rights approach and women perceive it as dehumanized [56]. This fact leads to an objectification of the victims [39, 55], they describe how their subjectivities are being reduced to a monetary transaction in such interventions. Something problematic in the sense that reparation should be accompanied by a psychosocial approach that is absent in these cases, as moral and symbolic dimensions that could have improved their reparation process towards an integral one.

*(...) they called my husband, they gave him a reparation of ten million pesos, because you know that it is given a check for each person, I always said that money is not everything because when they gave me the reparation that was not a huge amount of money. I shouted, cried, and said: until here they come [the reparation process]; how come a human being is going to be valued for x quantity? For seventy million pesos? Three people were repaired for that miserable thing; I prefer them with me and don't receive a cent* (Interview 11).

Negative structural dimensions as socio-economic precariousness are a constant; poverty conditions, marginalization, social exclusion and an institutional framework that does not properly articulate processes are evidenced. Therefore, a sociopolitical context of extreme vulnerability remains, and the psychosocial approach is not guaranteed. Social inequality frames the unsustainability of the reparation process as a recurrent fact, considering that psychosocial approach is not properly entailed, referring mostly to indemnification by itself. In this sense, psychosocial interventions that do not involve structural aspects can generate pathologizing processes, fragmenting and reducing social phenomena into individual realities [3, 15, 16, 35, 37, 38].

In a few cases, it is noticed that some of the State Interventions (GT) in relation to Measures of PR (OT) are found throughout Interventions that strengthen women’s capacities and resources (BT). These projects facilitate empowerment improving the community’s development from below and the social tissue reconstruction [14, 29, 30].

*We made the workshop with women’s training school in politics and peace in 2011. Now this year [2018] we also did women’s training in politics and peace, to work on women’s advocacy in a political way since we are now in the special constituency (*Interview 6).

*There have been meetings of care work, (...) and in the pedagogical meetings we revise all the themes of peace pedagogy, which was quite a subject matter that people were unaware of.* (Interview 6).

Women value PR that enables capacity building, as it allows them to acquire and strengthen skills for political participation towards peacebuilding and new comprehensions about the conditions of care work based on a differential gender approach [2, 35, 44–46]. In relation to the scarce PR experiences that were found, they permitted them to actively participate towards integral reparation, at strengthening their individual and collective resources and knowledge regarding a human rights approach.

Amongst the experiences shown on PR (OT), it was evident in isolated stories the presence of Participatory Social Projects (BT) that enable collective reparation to elaborate and mitigate psychosocial damages in communities. In this way, eliciting memory through art was an instrument for moral and symbolic reparation for peacebuilding in Montes de María. They show prosocial behavior and a strong sense of community as they are compromised towards granting the members needs as well as strengthening social networks in their region [6–9]. Striving to work for the culture and peace, they recognize that their regions’ needs have not been properly addressed.

*( … ) a community kiosk, the community kiosk has had good use, there have been memory workshops and there we have a photograph of each of the twelve people who were victims, photographs of the “Tamarindo tree”. That one is a place of memory, there the village’s Campesinos meet, we do activities, there also was created a reparation mentoring school* (Interview 12).

*(...) The symbolic part was phenomenal because the case was also published in the press and in some way through the press there are many people that also get to know. As we published on Facebook, many who didn’t have any knowledge about the massacre or that have forgotten because time had passed, they found out what was happening, and the aim is that this don’t happen ever again* (Interview 12).

These interventions involve their citizenships and community resources in order to contribute to their community empowerment. They are also developed from a community-based approach are more sustainable and aids the rebuilding process of the fragmented social tissue. However, most themes found in the women’s experiences are related to isolated humanitarian assistance measures. Such interventions when unaccompanied by PR, leads to an incomplete intervention. These implemented measures lack the required integrality towards Human Rights guarantee and the recognition of groups that are historically conditioned by inequality and marginalization; negating protection and social solidarity, which reproduces dehumanization through objectification and pathologization processes [39, 55, 56].

### State abandonment: agency as a response

Narratives expressed psychosocial damage caused by victimizing acts that have not been overcome and repaired by the State. Women recognize that they need emotional support in order to overcome psychosocial trauma, which requires PR. Women still experience fear, sadness, depression, resentment, stigmatization, discrimination, marginalization, social exclusion, post-traumatic stress, defeatedness, difficulties to forgive and reconcile and grieving amongst other negative feelings and emotions associated with the lack of opportunities and psychosocial accompaniment processes. Themes as Differential approach (BT), and Psychosocial rehabilitation demands (BT) are connected towards a Psychosocial Unresolved Damage (OT) at the Individual, familiar and community levels (BT), uncovering a negative impact in the women’s experiences due to a lack of Psychosocial Approach in the Law’s implementation.

*Sometimes the reconciliation theme is very difficult, to heal that wound when you has not overcome that type of mourning, it’s so difficult at times. At least I have capacitated myself; it’s difficult for me to forgive those actors [of conflict] because I believe that words asking for forgiveness does not heal, you must heal with psychological help, because the damage is multiple, the damage has been from many years (...) I haven’t received that accompaniment that allows me to approach this emotional situation with myself, maybe face that reality that I lived* (Interview 8).

Violence continues in their territories as campesino communities are threatened by illegal armed groups at claiming restitution and reparation measures, aspects that establish limitations towards mitigating pain and granting satisfaction and non-guarantee measures in relation to integral reparation. It is evidenced that the violent social conditions are strongly present in their stories and they do not have the necessary tools for reparation.

*When the claim process occurs with Law 1448, the land restitution unit is created here for the first time, it was opened here. As Campesinos we began to claim the land and then people started to threaten the Campesino people, to threaten those of us who are at the forefront, accompanying and advising the Campesino people to make their claim* (Interview 8).

It is noticed that women describe that they have received the negative consequences of state abandonment; violence, discrimination, a lack of opportunities, unemployment and social inequality to a greater extent than men. After the victimizing events, many women struggle in order to adapt to caring tasks relating to their homes and children without the masculine role, feeling overwhelmed with survival duties. These realities claim for the differential approach of the VL psychosocial interventions regarding women’s necessities towards reaching a social equilibrium upon the differential affectations of armed conflict framed in gender structures [35, 43–46, 69]. The multiple implementation limitations are shown through their narratives, as women lack PR with a gender differential approach.

*A woman alone is very difficult, it’s very difficult for a woman to arrive with three children ( … ) when as a woman you have your husband, you are used to him being the one who solves everything ( … )* (Interview 2).

*They are threatening me, they are telling me, still continue, if I had been a man, that person would not mess with me, because we women are always as popular knowledge says ´the famous weak sex´, but no, we are not weak, at least I am a woman of ax and knife* (Interview 2).

*I have never had the opportunity to have a job, for me to help my husband, my family, it is one of the things that I see amongst women that there is a lot of unemployment, there are many unemployed women because they do not give us the opportunity* (Interview 10).

Faced with State Abandonment (GT) is found Revictimization (BT), and subsequently, Agency as a response to abandonment (OT), becoming a resistance and coping mechanism. They recognize that integral reparation process has exceeded the State’s capacity towards implementation; they feel left on their own in the quest for survival without adequate support. Also, as they experienced many difficulties at accessing the Law’s protection, assistance and reparation measures, they even stopped trying to get access to reparation, or in other cases found no answers from the State. They indicate that their humanity, dignity and Human Rights should be protected through the Law’s implementation, but rather encounter no actions or disintegrated ones that leave a negative impact of revictimization on them.

*I perceive that there are many needs and nobody helps us ( … ) They say that it will come, that someday they will help, but until now no, nothing, nothing, we keep struggling every day, but the government no, the government doesn't give us anything* (Interview 2).

*Is going to be two thousand and twenty and there will be people who did not receive anything ( … ) I am still struggling, I come and go. But I no longer have the desire to fight … the State has not been able to attend so many people, the people of Carmen de Bolívar, with what they suffered in here (*Interview 5).

*(...) as a woman, I feel re-victimized by the State (*Interview 8).

In this way, the general perception is of State abandonment, showing a socio-economic and political intervention gap as many individuals and communities have been waiting for several years and are still expecting for reparation. So, instead of providing truth, justice, reparation and non-repetition guarantees towards an integral reparation, it implies further re-victimization processes by the State, as the conflict’s roots based on social inequality and PR measures are not properly addressed. Meanwhile, agency as an answer from women towards State abandonment (OT) is evidenced in Self-management (BT) and Community Resources (BT) resources that they strive to overcome the negative impacts and limitations of armed conflict.

*The psychological help I had was because I financed it, the State didn’t told me: look we are going to put you here, here you have the transportation, here you have this for you to go to your psychology aid, no* (Interview 1).

*We have to move forward struggling for life, for our children and for the community, through our life story we can give an example of strength to help other people, make them feel that they are not the only ones who lived difficult moments( … )* (Interview 12).

They are certainly not passive actors, as they struggle on their own against the negative socio-economic conditions, the prolongation of psychosocial trauma and revictimization processes perpetuated by the State. Now, in some cases they have had to finance their psychological processes, as the Law did not respond to those moral and symbolic dimensions of reparation. As well as they are making efforts to improve their opportunities related to the precarious socioeconomic conditions. Women instead are resilient, have a proactive attitude, solidarity and creativity at believing that there are many reasons to continue working for a better life for themselves, their children and their communities; as well as achieving their collective interests with the support of third-party organizations.

*( … ) I still continue in this struggle, because it’s a daily struggle, a struggle that we have with the State because that is a [matter] of institution, here the only culprit is the institution, we didn’t have a reason to live all of this, but in this moment I have kept moving forward, I have not stayed like “poor me”, like the victim, the displaced women that receive humanitarian aids or indemnification. I have overcome it, in this moment I am in third semester of social work, I have trained myself, it’s never too late for studying and I studied customer service, and those studies have helped me obtain some jobs and get through my life with my childre*n (Interview 8).

*( … ) That motivated me to come back, but also to strengthen women processes over there because the theme is unknown, that’s why we wanted to carry out our proposal called “New voices for territorial peacebuilding” in the lower zone of Carmen de Bolívar, because we consider that although all that we suffered in the conflict, it is a zone that has not been properly attended; so I believe is not late, that we can continue strengthening an interesting process with women and their families so that they can grant their rights and organize in the collective level to present their proposal, from the local and institutional, so that the departmental and national institutions can take a glance towards this zone and truly start a strengthening and participatory process for women* (Interview 9).

*Women are the figure that has maintained unity, and maybe we maintain peace because we tell our children and friends: “no, that’s not the pathway, maybe things are going to happen for us”* (Interview 1).

A strong sense of community and political participation found in the micro-systemic, meso-systemic and macro-systemic levels [15, 29, 30, 70] are important ingredients in the process of self-reparation after they recognized State abandonment. They have constituted organized community work in which women empower each other to demand their rights, strengthen their individual and community capacities and construct peace in Montes de María. They acknowledge the psychosocial approach potential and demand the reparation of their rights from a Human Rights, critical and communitarian approach [2, 26, 35, 37]. The necessary participatory processes required on PR are being overtaken with their own individual and collective resources, towards State absence.

## Discussion

The VL framed in a sociopolitical paradigm, in its design aims to grant truth, justice, and integral reparation. Psychosocial rehabilitation should be found throughout all the implementation phases with concrete actions and processes related to material and symbolic measures [2, 4]. Besides, the VL proposes to transform the victims’ structural conditions through the community’s participation and empowerment, giving a significant role to the knowledge and practices led by the victims, as occurred in its design [6, 14, 31–33]. However, results show that women victims of Montes de Maria do not recognize a humanizing, properly articulated psychosocial approach, which recognizes their resources and capacities, despite the multiple collective initiatives that they have carried out to guarantee psychosocial well-being in their territories.

In relation to this, it can be argued that the most recurrent type of State interventions experienced by women is based on the Non-sociopolitical or directive intervention paradigm which does not understand the structural roots of the Colombian conflict, neither transforms those victims’ structural conditions; or promote their active participation in reparation processes [14, 19]. Therefore, as a sociopolitical context of extreme vulnerability remains, and the psychosocial approach is not guaranteed [23, 29, 30, 35, 37], their constitutional rights are further violated from a non-socio political paradigm in contrary to the Law’s spirit of Integral Reparation [2, 3, 16].

Furthermore in contrast to the VL, in the case of women victims in Montes de María, non-sociopolitical or directive interventions per se, that do not involve structural aspects, generate pathologizing processes [39, 55], fragmenting and reducing social phenomena into individual realities [23, 28–30, 38]. Interventions should include individual, micro-systemic levels, as well as relational and community intervention that relate to the meso-systemic and macro-systemic levels towards the systemic or holistic approach, however the results show vast limitations to its integrality aims [2, 29, 30, 43, 70]. Humanitarian assistance and indemnification, which refer to the reparation’s material dimension, are disintegrated from the VL intersectoral and integral perspective. The Psychosocial approach in its moral and symbolic dimensions are not properly entailed and does not attend to the victimization’s contextual aspects, depoliticizing and dehumanizing victims [39, 55, 56]. In order to grant an integral reparation, it is important to reinforce the evaluation on the effectiveness of VL’s psychosocial and mental health care programs. The lack of evaluation processes is affecting the restoration of their rights [24].

Equally important in opposition with the VL is the fact that due to illegal groups that force their presence in the territories [50] and the structural violence that women encounter, they are found in extreme poverty conditions, in a struggle to satisfy basic daily necessities which shows the precariousness that remains in the communities without effective social policies that limits a neoliberal socio-political system [29, 30, 34, 51–53, 56]. The multiplicity of vulnerabilities that are configured and are not addressed by the Law implementation provides an Unconstitutional State of affairs that persists, although the Constitutional Court Sentence T-025 of 2004 indicated that these conditions had to be overcome by the internally displaced communities with pertinent State interventions.

Nevertheless, a couple of cases narrated PR experiences with differential approaches. These PR processes involve structural aspects in two ways. First, peace pedagogy for the strengthening of political participation as essential for the construction of a positive peace towards more democratic societies [3, 13, 16, 34, 35, 50]. Secondly, political participation situated from a gender approach, articulating pedagogical processes with women’s care work. Such citizenship practices are shown to be essential in post-war settings and women’s political participation [15, 38, 44–46]. A mentioned case, experienced community PR processes in moral and symbolic dimensions. The interviewee was emphatic on how this type of intervention allowed recognition of the sociopolitical nature of the victimizing acts and helped to repair the fragmented social tissue [6, 14, 29, 30, 49]. Both aspects permit us to affirm that when PR interventions occur from a socio-political paradigm, favoring social transformations that mobilizes victimizing acts towards community empowerment, the VL integrality aim is achieved.

Unfortunately, since most cases have not received PR, there is unrepaired psychosocial trauma that is present at the individual, familiar and community levels, which reflect the second global theme: state abandonment. In this sense, as an appropriate measure, the VL was extended by the Constitutional Court until 2030 in correspondence with the victim’s needs and revictimization processes found in this study. Certainly, one of the approaches on which further implementation and evaluation is required is a transversal perspective of the psychosocial approach to achieve the Law’s goals [3, 16, 35, 38].

Faced with this situation, women victims have managed individual and community resources to promote political participation, psychosocial processes and community development from below as a resistance to the structural violence and precariousness they have encountered [43, 44, 46, 47]. These agency responses are identified by them as an intended contribution to peacebuilding in their region. Throughout these collective actions it can be argued that women’s sense of community enables social justice and peacebuilding as well as the reparation of the fragmented social tissue of Montes de María that the State is not providing [3, 6, 16, 23, 34].

Moreover, they identified this agency as a response to the negative psychosocial consequences that the prolongation of armed conflict implies on revictimization processes due to an absence of non-repetition guarantees and satisfaction measures [50]. It is important to recognize the leadership that they have developed amid the socioeconomic and political violence they have faced as it has been the mechanisms by which they have guaranteed the well-being of them, their families and communities [15, 43–46, 53]. To sum up, these women initiatives and leaderships are key processes to build community-based PR in Montes de Maria, which could guarantee a socio-political intervention through participation and empowerment. This in cooperation with psychosocial professionals from third party organizations who in a horizontal dialogue with popular knowledge could facilitate the construction of strategies and programs that contribute to the VL’s integral implementation and avoid revictimization.

## Conclusion

Due to the propensity of non-sociopolitical interventions in Montes de Maria, in opposition to the VL sociopolitical perspective, further monitoring and evaluation processes of the psychosocial approach, specifically of PR, are required for a more efficient response to the communities’ needs regarding the execution of State’s resources. PR has not been experienced by women, as humanitarian assistance and some cases of indemnification have not been articulated with symbolic and collective actions, which does not respond to integral reparation. Revictimization and State abandonment persist, on a structural violence basis towards unsolved psychosocial trauma in which women have replied with agency and political participation for peacebuilding, contributing to the community’s development in a resilient way.

In this sense, the psychosocial interventions found in these women communities serve at recognizing the role they may have as advisors and collaborators of individual and collective reparation implementation, to respond effectively to the magnitude of the victim’s demands as they contributed to the construction of the law. Such intervention timing and resources gap could be improved with the active interventions that communities develop on their own. The community capacity that women reflect enables the VL to revise the strengthening of collective reparation in harmony with the territorial actions with which communities make resistance. Further articulation of the VL collective intervention route, with a focused territorial and differential approach that meets their needs, as well as the unexplored women’s peacebuilding resources that strengthen and support their political participation skills, are suggested paths to develop a coherent and effective PR and public health policy implementation for women victims in Montes de Maria towards reaching a social equilibrium.

## Data Availability

The datasets generated and/or analyzed during the current study are not publicly available due to protection and safety issues of the participating women whose narratives would allow their identification but are available from the corresponding author on reasonable request.

## Abbreviations

PR: Psychosocial Rehabilitation
VL: Victims Law
GT: Global Theme
OT: Organizing Theme
BT: Basic Theme

## References

[1] Red Nacional de Información. Registro Único de Víctimas. Colombia; 2020. Available from: https://www.unidadvictimas.gov.co/es/registro-unico-de-victimas-ruv/37394

[2] Congreso de la Repùblica de Colombia (2011). Law 1448 of 2011.

[3] Moreno MA, Moncayo JE, Moncayo JE, Díaz Á (2015). Abordaje psicosocial. Consideraciones conceptuales y alternativas de análisis en el escenario de atención a víctimas del conflicto armado. III Libr Psicol Soc crítica Psicol Soc crítica e Interv Psicosoc Reflexiones y Exp Investig.

[4] Unidad para la Atención y la Reparación Integral a las Víctimas. Unidad para la atención y reparación integral a las víctimas. Available from: https://www.unidadvictimas.gov.co/. Accessed 15 July 2020.

[5] López-López W (2020). A Multidimensional &amp; Dynamic Perspective of Research &amp; Intervention in Peace Psychology. Peace Psychol.

[6] Arango C (2008). Pobreza, participación y desarrollo comunitario en el Estado de Bienestar. Psicol comunitaria la Convivencia.

[7] Long A, Perkins D (2007). Community social and place predictors of sense of community: a multilevel and longitudinal analysis. J Community Psychol.

[8] Mak W, Cheung R, Law L (2009). Sense of community in Hong Kong: relations with community-level characteristics and residents’ well-being. Am J Community Psychol.

[9] McMillan D, Chavis D (1986). Sense of community: a definition and theory. J Community Psychol.

[10] Trujillo SP, Trujillo N, Ugarriza JE, Uribe LH, Pineda DA, Aguirre-Acevedo DC (2017). How empathic are war veterans? An examination of the psychological impacts of combat exposure. Peace Confl J Peace Psychol.

[11] O’Rourke C, Buckley-Zistel S, Stanley R (2012). Transitioning to what? Transitional justice and gendered citizenship in Chile and Colombia. Gend transitional justice Gov ltd statehood Ser.

[12] Programa de las Naciones Unidas para el Desarrollo (2011). El ABC de la ley de víctimas. Hechos Paz.

[13] Roberts BR. The new social policies in Latin America and the development of citizenship: an interface perspective: Wageningen; 2001. Available from: http://dev.lanic.utexas.edu/project/etext/llilas/claspo/workingpapers/newsocpolicies.pdf

[14] Montenegro M. Conocimientos, Agentes y Articulaciones. Una mirada situada a la Intervención Social. Athenea Digit Rev Pensam e Investig Soc. 2001; Available from: https://atheneadigital.net/article/view/n0-montenegro/17.

[15] Cadena-Camargo Y, Krumeich A, Duque-Páramo MC, Horstman K. “We just been forced to do it”: exploring victimization and agency among internally displaced young mothers in Bogotá. Confl Health. 2019;13 Available from: https://conflictandhealth.biomedcentral.com/articles/10.1186/s13031-019-0205-1#citeas. 10.1186/s13031-019-0205-1 PMC6547547 31171933

[16] Moreno-Camacho MA, Díaz-Rico ME (2016). Posturas en la atención psicosocial a víctimas del conflicto armado en Colombia. El Ágora USB.

[17] López-López W, Correa-Chica A, Sierra-Puentes MC, Castañeda Polanco JG, Fernández Miranda G, Duran Jaramillo M, et al. Children’s Conceptualizations of Forgiveness, Reconciliation, and Peacebuilding in the Context of Armed Conflict. In: Balvin N, Christie D, editors. Child Peace From Res to Action. Cham: Springer Open; 2020. p. 203–15.

[18] Davis S. Forgiveness and reconciliation: essential to sustaining human development. In: Kalayjian A, Paloutzian RF, editors. Forgiveness Reconcil. Cham: Springer; 2009.

[19] Corvalán J (1996). Los paradigmas de lo social y las concepciones de intervención en la sociedad.

[20] Presidente de la Repùblica de Colombia. Decree 4800 of 2011. Colombia: Congreso de la Republica de Colombia; 2011.

[21] López-López W, Sandoval Alvarado G, Rodríguez S, Ruiz C, León JD, Pineda-Marín C, et al. Forgiving former perpetrators of violence and reintegrating them into Colombian civil society: noncombatant citizens’ positions. Peace Confl J Peace Psychol. 2018;c24(2):201–15. 10.1037/pac0000295.

[22] Mullet E, Neto F (2009). Lay People’s views on intergroup forgiveness. Peace Rev.

[23] Nelson G, Prilleltensky I (2010). Community psychology. Purs lib well being.

[24] Hewitt-Ramírez N, Juárez F, Parada-Baños AJ, Nuñez-Estupiñán X, Quintero-Barrera L (2020). Efficacy of a primary care mental health program for victims of the armed conflict in Colombia. Peace Confl J Peace Psychol.

[25] Morin E (1999). Los Siete saberes necesarios para la educación del futuro.

[26] Aya- Angarita SL, Laverde-Gallego D (2016). Comprensión de perspectivas psicosociales en Colombia. Rev Divers en Psicol.

[27] Sacipa S, Tovar C, Sarmiento L, Gómez A, Suárez MP. La psicología política en Colombia. Les Cah Psychol Polit. 2013; Available from: http://lodel.irevues.inist.fr/cahierspsychologiepolitique/index.php?id=2559.

[28] Hodgetts D, O’Doherty K, O’Doherty K, Hodgetts D (2019). Introduction: applied social psychology- an evolving tradition. SAGE Handb Appl Soc Psychol.

[29] Montero M (2004). Relaciones Entre Psicología Social Comunitaria, Psicología Crítica y Psicología de la Liberación: Una Respuesta Latinoamericana. Psykhe.

[30] Montero M (2004). Introducción a la psicología comunitaria. Desarrollo, conceptos y procesos.

[31] de Sousa B, Gandarilla JG (2009). Una epistemología del sur: la reinvención del conocimiento y la emancipación social.

[32] De Sousa B (2010). Descolonizar el saber, reinventar el poder.

[33] Fals Borda O (1959). Acción comunal en una vereda colombiana.

[34] Galtung J (1990). Cultural Violence. J Peace Res.

[35] Weber S (2019). Trapped between promise and reality in Colombia’s Victim’s Law: reflections on reparations, development and social justice. Bull Lat Am Res.

[36] Villa JD (2012). La acción y el enfoque psicosocial de la intervención en contextos sociales: ¿podemos pasar de la moda a la precisión teórica, epistemológica y metodológica?. El Ágora USB.

[37] Lugo V, Sánchez PV, Rojas C (2018). La restauración con sobrevivientes del conflicto armado en Colombia: una propuesta de acción psicosocial. Rev Eleuthera.

[38] L Wirtz A, Pham K, Glass N, Loochkartt S, Kidane T, Cuspoca D, et al. Gender-based violence in conflict and displacement: qualitative findings from displaced women in Colombia. Confl Health 2014;8. Available from: https://conflictandhealth.biomedcentral.com/articles/10.1186/1752-1505-8-10#citeas 10.1186/1752-1505-8-10 PMC4115473 25076981

[39] Fassin D, Vásquez P (2005). Humanitarian exception as the rule: the political theology of the 1999 Tragedia in Venezuela. Am Ethnol.

[40] O’Rourke C (2015). Feminist scholarship in transitional justice: a de-politicising impulse?. Womens Stud Int Forum.

[41] Villa JD, Barrera D, Arroyave L, Montoya Y. Acción con daño: del asistencialismo a la construcción social de la víctima. Mirada a procesos de reparación e intervención psicosocial en Colombia. Univ Psychol. 2017;16(3):1–13.

[42] Delgado BM (2015). Las víctimas del conflicto armado colombiano en la Ley de Víctimas y Restitución de Tierras: apropiación y resignificación de una categoría jurídica. Perfiles Latinoam.

[43] Centro Nacional de Memoria Histórica. Las Resistencias (2018). Género y Mem Histórica Balanc la Contrib del CNMH al esclarecimiento histórico.

[44] Bouvier VM. El Género y el Papel de las Mujeres en el Proceso de Paz de Colombia. Nueva York; 2016. Available from: https://www.usip.org/publications/2016/11/gender-and-role-women-colombias-peace-process

[45] Salcedo DM. Género, derechos de las víctimas y justicia transicional: Retos en Colombia. Rev Paz y Conflictos. 2013;6 Available from: https://revistaseug.ugr.es/index.php/revpaz/article/view/813.

[46] Ruta Pacífica de las Mujeres. Una Verdad que Repare. In: Aportes desde la propuesta metodológica de la Ruta Pacífica de las Mujeres para la Comisión de Esclarecimiento de la Verdad. Bogotá; 2018. Available from: www.rutapacifica.org.co.

[47] Sarmiento LC. Mulheres e narrativas de reparação : a categoria gênero problematizando a justiça transicional colombiana: Universidade de Brasília; 2018. Available from: https://repositorio.unb.br/handle/10482/34730

[48] Andrade Salazar JA, Alvis Barranco L, Jiménez Ruiz LK, Redondo Marín MP, Rodríguez González L (2016). Secuelas Psicológicas de la Guerra en Mujeres Forzadas a Desplazarse Psychological After-effects of War in Displaced Women. Rev Int Psicol.

[49] Tol W, Stavrou V, Greene C, Mergenthaler C, Ommeren M, García C. Sexual and gender-based violence in areas of armed conflict: a systematic review of mental health and psychosocial support interventions. Confl Health. 2013;7 Available from: https://conflictandhealth.biomedcentral.com/articles/10.1186/1752-1505-7-16#citeas. 10.1186/1752-1505-7-16 PMC3750365 23915821

[50] Ramírez-Orozco M. Aproximación bibliográfica a la construcción de la paz en Colombia. Rev la Univ La Salle. 2014:23–43 Available from: https://ciencia.lasalle.edu.co/ruls.

[51] Álvarez S (2005). Los discursos minimalistas sobre las necesidades básicas y los umbrales de ciudadanía como reproductores de la pobreza. Trab y Prod la Pobr en Latinoamérica y el Caribe Estructuras, Discursos y Actores.

[52] De Zubiría S (2015). Dimensiones políticas y culturales en el conflicto colombiano.

[53] Rodríguez L (2020). ‘We are not poor things’: territorio cuerpo-tierra and Colombian women’s organised struggles. Fem Theory.

[54] Vargas LL (2018). La reparación integral a las víctimas del conflicto armado en el sur del departamento del Huila en el marco de la Ley de Víctimas. Diálogos de Saberes.

[55] Fassin D, Gomme R. Humanitarian Reason: A Moral History of the Present. 1st ed. Oakland: University of California Press; 2012.

[56] Butler J (2010). Introducción: Vida precaria, vida digna de duelo. Marcos Guerr Las vidas lloradas.

[57] Creswell J, 2nd Edition (2007). Five Qualitative Approaches to Inquiry. Qual Inq Res Des Choos among five approaches.

[58] Lambert C (2006). Edmund Husserl: la idea de la fenomenología. Teol y vida.

[59] Grupo de Memoria Histórica (2011). Mujeres y guerra. Víctimas y resistentes en el Caribe colombiano.

[60] Congreso de la Repùblica de Colombia (2006). Law 1090 of 2006.

[61] Caillaud S, Flick U, Barbour RS, Morgan DL (2017). Focus Groups in Triangulation Contexts. A New Era Focus Gr Res.

[62] Chase SE, Denzin N, Lincoln Y (2005). Narrative Inquiry: Multiple Lenses, Approaches, Voices. SAGE Handb Qual Res.

[63] Flick U. An introduction to qualitative research. London: Sage Publications; 2006.

[64] Jovchelovitch S, Bauer M. Narrative interviewing. In: Bauer MW, Gaskell G, editors. Qual Res with Text, Image Sound A Pract Handb. Longon: Sage ; 2000. Available from: 10.4135/9781849209731

[65] Pinnegar S, Daynes JG, Clandinin DJ (2007). Locating Narrative Inquiry Historically: Thematics in the Turn to Narrative. Handb Narrat Inq Mapp a Methodol.

[66] Attride-Stirling J (2001). Thematic networks: an analytic tool for qualitative research. Qual Res.

[67] Flick U, Flick U, Kardoff E, Steinke I (2004). Triangulation in qualitative research. A companion to Qual res.

[68] Tong A, Sainsbury P, Craig J (2007). Consolidated criteria for reporting qualitative research (COREQ): a 32-item checklist for interviews and focus groups. Int J Qual Health Care.

[69] Centro Nacional de Memoria Histórica (2018). Memorias plurales: experiencias y lecciones aprendidas para el desarrollo de los enfoques diferenciales en el Centro Nacional de Memoria Histórica:balance de la contribución del CNMH al esclarecimiento histórico.

[70] Bronfenbrenner U (1987). La ecología del desarrollo humano.

